# Chronic Vitamin E Deficiency Dysregulates Purine, Phospholipid, and Amino Acid Metabolism in Aging Zebrafish Skeletal Muscle

**DOI:** 10.3390/antiox12061160

**Published:** 2023-05-26

**Authors:** Trent D. Henderson, Jaewoo Choi, Scott W. Leonard, Brian Head, Robyn L. Tanguay, Carrie L. Barton, Maret G. Traber

**Affiliations:** 1 Linus Pauling Institute, College of Public Health and Human Sciences, Oregon State University, Corvallis, OR 97331, USA; hendetre@oregonstate.edu; 2 Linus Pauling Institute, Oregon State University, Corvallis, OR 97331, USA; jaewoo.choi@oregonstate.edu (J.C.); scott.leonard@oregonstate.edu (S.W.L.); bhead@exponent.com (B.H.); 3 Sinnhuber Aquatic Research Laboratory, Environmental Health Sciences Center, Oregon State University, Corvallis, OR 97331, USA; robyn.tanguay@oregonstate.edu (R.L.T.);

**Keywords:** α-tocopherol, sarcopenia, lipid peroxidation, metabolomics, *Danio rerio*

## Abstract

Muscle wasting occurs with aging and may be a result of oxidative stress damage and potentially inadequate protection by lipophilic antioxidants, such as vitamin E. Previous studies have shown muscular abnormalities and behavioral defects in vitamin E-deficient adult zebrafish. To test the hypothesis that there is an interaction between muscle degeneration caused by aging and oxidative damage caused by vitamin E deficiency, we evaluated long-term vitamin E deficiency in the skeletal muscle of aging zebrafish using metabolomics. Zebrafish (55 days old) were fed E+ and E− diets for 12 or 18 months. Then, skeletal muscle samples were analyzed using UPLC-MS/MS. Data were analyzed to highlight metabolite and pathway changes seen with either aging or vitamin E status or both. We found that aging altered purines, various amino acids, and DHA-containing phospholipids. Vitamin E deficiency at 18 months was associated with changes in amino acid metabolism, specifically tryptophan pathways, systemic changes in the regulation of purine metabolism, and DHA-containing phospholipids. In sum, while both aging and induced vitamin E deficiency did have some overlap in altered and potentially dysregulated metabolic pathways, each factor also presented unique alterations, which require further study with more confirmatory approaches.

## 1. Introduction

Sarcopenia, as defined by the European Working Group on Sarcopenia in Older People, consists of low muscle strength, poor muscle quantity/quality, and poor physical performance [1,2]. Furthermore, sarcopenia has been associated with higher rates of falls and fractures, leading to increased incidence of hospitalizations, morbidity, and mortality [3,4,5]. Loss of muscle strength occurs with aging and may be a result of oxidative damage and potentially inadequate protection by antioxidants, such as vitamin E [6]. In contrast, increased skeletal muscle mass and bone density are associated with higher vitamin E status, as shown in the EPIC-Norfolk longitudinal study [7]. Similarly, higher dietary intakes of vitamin E and fats in Japanese people greater than 60 years of age were associated with a low prevalence of sarcopenia [8]. Additionally, a 30-year prospective cohort study ((29,092 participants in the ATBC (Alpha-Tocopherol, Beta-Carotene Cancer Prevention) study) found that higher baseline serum α-tocopherol was associated with lower overall mortality [9].

With aging, there is a need for sarcopenia prevention, and the above studies suggest increased vitamin E intake may be beneficial. Vitamin E deficiency was recognized early in its discovery to cause not only neuronal but also muscle damage [10,11], but less is known about the mechanism between vitamin E and sarcopenia. To link vitamin E and sarcopenia, we sought to explore whether there is an interaction between muscle degeneration caused by aging and oxidative damage caused by vitamin E deficiency. We propose that adult vitamin E-deficient zebrafish are an excellent model for studying sarcopenia in that they have shown severe, progressive muscle damage when fed a vitamin E-deficient diet for a prolonged period [12] and have also shown evidence of sarcopenia [13]. Zebrafish have a reported mean lifespan of 32 months [14]; therefore, we studied zebrafish fed from 55 days up to 12 and to 18 months on our defined diets with or without vitamin E. This length of time was chosen because there is only a low rate of mortality up to 18 months in zebrafish [14].

We hypothesize that a prolonged insufficient dietary intake of vitamin E, specifically α-tocopherol, may cause the dysregulation of the skeletal muscle cellular metabolism, similar to previous reports examining vitamin E-deficient zebrafish embryos [15,16,17] or zebrafish brains [18,19]. Thus, we used semi-quantitative mass spectrophotometric techniques (metabolomics) to identify metabolic relationships between insufficient vitamin E intake and skeletal muscle degeneration as a result of vitamin E deficiency and as a result of aging. Such an outcome would lend support to advocates of modifiable risk factors such as vitamin E intakes, not only as a means to attenuate age-related skeletal muscle decline such as sarcopenia but other age-related diseases as well [9].

## 2. Materials and Methods

### 2.1. Materials and Reagents

Supplies were obtained as follows: ammonium formate (Optima; ThermoFisher, Carlsbad, CA, USA), formic acid (Optima), tris (2-carboxyethyl) phosphine hydrochloride (TCEP; MilliporeSigma, St. Louis, MO, USA), *N*-ethylmaleimide (NEM; MilliporeSigma); ethylenediaminetetraacetic acid (EDTA; MilliporeSigma), 5-sulfosalicylic acid (SSA; MilliporeSigma), and zirconium oxide beads (Next Advance; Troy, NY, USA). All other reagents and solvents were of analytical grade.

### 2.2. Zebrafish Husbandry and Dietary Conditions

The Institutional Animal Care and Use Committee (IACUC) of Oregon State University approved the protocol for the study (ACUP #5068). Tropical 5D strain zebrafish were reared in the Sinnhuber Aquatic Research Laboratory at Oregon State University under standard laboratory conditions of 28 °C on a 14 h light/10 h dark photoperiod according to standard zebrafish breeding protocols [15]. At 55 days post-fertilization, zebrafish were randomly allocated to one of two experimental diets, vitamin E deficient (E−) or sufficient (500 mg *RRR*-α-tocopheryl acetate/kg diet, E+), as previously described [15,20,21]. The adult fish consumed their respective diets for 12 or 18 months before euthanasia by cold exposure in ice water. Then, muscle tissue with attached skin was removed, frozen in liquid nitrogen, and stored at −80 °C until analysis.

### 2.3. Muscle Extraction

Zebrafish skeletal muscles and attached skin (n = 10 per age group, 12 or 18 months) were analyzed from fish (sex not determined) fed either E− or E+ diets. Samples (approximately 50 mg) were dissected and weighed individually. Samples were loaded into Precellys Lysing Kit bead blender tubes with pre-supplied beads, and 300 microliters solvent (methanol: water, 80:20 *v*/*v*) was added for homogenization (Precellys 24 Tissue Homogenizer, www.bertin-instruments.com, URL (accessed 24 May 2023). Samples were homogenized (5500 rpm) for three 30 s cycles with 15 s pauses between cycles. The homogenates were then centrifuged at 21,130× *g* for five minutes. Supernatants (exactly 200 μL) were transferred to clean tubes, dried under nitrogen, then reconstituted with solvent (acetonitrile: water, 1:1 *v*/*v*). To maintain consistent concentrations between samples, solvent volumes were then adjusted based on the starting weight of the sample (1:5, sample to volume), then transferred individually to new tubes and stored at −80 °C until analysis via LC-MS/MS. Quality control (QC) samples (n = 5) were generated by pooling 10 μL aliquots from each muscle sample extract and were analyzed with the muscle samples. Blanks that contained only solvent were also analyzed. Samples were analyzed in a single run, and data were adjusted for QC value responses.

### 2.4. LC-MS/MS for Metabolomic Analysis

Chromatography was performed using a Shimadzu Nexera system (Shimadzu; Columbia, MD, USA) coupled to a high-resolution hybrid quadrupole-time-of-flight mass spectrometer (TripleTOF^®^ 5600; SCIEX; Framingham, MA, USA). An HILIC column was used for LC analysis. The separation was carried out using a 2.1 × 150 mm SeQuant ZIC-pHILIC (5 µm, EMD Millipore, Billerica, MA, USA) with a 2.1 × 20 mm guard column. The flow rate was 0.2 mL/min, and the injection volume was 5 µL. The two mobile phases consisted of 10 mM ammonium acetate in acetonitrile: water (5:95, *v*/*v*) as mobile phase A and 10 mM ammonium acetate in acetonitrile: water (95:5, *v*/*v*) as mobile phase B. The gradient was as follows: an initial hold at 90% B for 1 min, followed by a gradient of 90–40% B in 18 min. The gradient was stepped back to 90% B at 20 min, and the column was equilibrated for 6 min. The column temperature was held at 45 °C, and the autosampler was kept at 10 °C.

The time-of-flight (TOF) mass spectrometer (MS) was operated with an acquisition time of 0.25 s and a scan range of 70–1000 Da. MS/MS acquisition was performed with collision energy set at 35 V and a collision energy spread of 15 V. Each MS/MS scan had an accumulation time of 0.17 s and a range of 40–1000 Da using information-dependent acquisition (IDA). The source temperature was set to 500 °C, and IonSpray voltage was set to 4.5 kV in positive ion mode and −4.0 kV in negative ion mode, respectively.

### 2.5. Quantitation and Statistical Analysis of α-Tocopherol in Tissues

Following storage at −80 °C, approximately 50 mg muscle tissue and attached skin was accurately weighed, and α-tocopherol concentrations were determined using high-performance liquid chromatography with electrochemical detection, as described in [22]. α-Tocopherol was quantitated by comparison to authentic α-tocopherol (MilliporeSigma). Statistical interactions between age and diet groups were calculated using 2-way ANOVA with Tukey’s multiple comparison tests (Prism 6.0, Graphpad, La Jolla, CA, USA). A cutoff for statistical significance was set at a *p*-value < 0.05.

### 2.6. Metabolomics Data Processing and Statistical Analyses

Raw MS files were imported and processed using the program PeakView Ver. 1.2 (Sciex). PeakView detects spectral features using XIC lists from our in-house library of metabolites consisting of IROA standards (IROA Technology, Bolton, MA; each defined by a unique chromatographic retention time (error < 10%), accurate mass (error < 10 ppm), MS/MS fragmentation (score > 70), and isotopic pattern (error < 20%)). In addition to the IROA database, the fragmentation spectra of all peaks were verified with data from Metlin [https://metlin.scripps.edu, URL (accessed 24 May 2023)] and HMDB [https://hmdb.ca, URL (accessed 24 May 2023)]. Each peak was integrated using MultiQuant Ver. 3.0.2 software (Sciex). Upon confirmation of peak areas in MultiQuant per individual metabolite and for all 20 samples, data were saved for export into Excel, where data were corrected using QC response data for each metabolite and combined before uploading to MetaboAnalyst 5.0. The data is provided in Table S1.

One-factor statistical analysis was used to create heatmaps and evaluate partial least squares–discriminant analysis (PLS-DA) and variable importance in projection (VIP) using MetaboAnalyst 5.0 software [http://www.metaboanalyst.ca, URL (accessed 24 May 2023)]. Exported data were uploaded from Excel, log transformed, and autoscaled upon upload to MetaboAnalyst. Autoscaling was chosen to assign equal importance to each respective metabolite [23]. Dendrogramatic clustering on the y-axis was based on Euclidean distance. MetaboAnalyst 5.0 software was also used to analyze the metabolic pathways in accordance with their differential metabolite responses. Upon upload, the data set was normalized by median, log transformation, and autoscaling prior to visualization of pathway analysis. Pathways were considered significant when impact > 0.3 and false discovery rate (FDR) < 0.05.

## 3. Results

### 3.1. Muscle Vitamin E Concentrations

**Figure 1:**
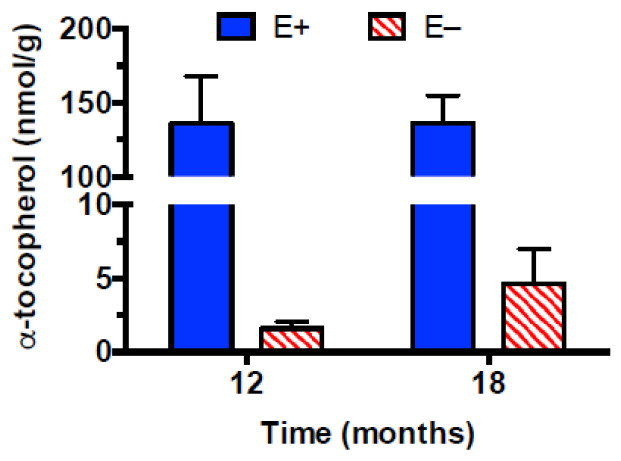
Muscle α-tocopherol concentrations. Zebrafish from the same cohort were fed a vitamin E-sufficient (E+, blue bars, n = 5 per group) or -deficient (E−, red striped bars, n = 5 at 12 months and n = 4 at 18 months) diet from age 45 days until 12 or 18 months. At the indicated times, fish were sacrificed, the muscles harvested, frozen, and kept frozen until analysis. α-Tocopherol skeletal muscle concentrations (mean ± SD) show a diet main effect (*p* ≤ 0.0001, no significant interaction or main effect of time).

Muscle α-tocopherol concentrations were analyzed to quantify and verify vitamin E deficiency. Muscles from the E+ fish contained about 45 times more α-tocopherol than those from the E− fish (Figure 1, main effect of diet, *p* < 0.0001, 2-way ANOVA), but neither the interaction nor the effect of time was significant. Thus, muscles from E− zebrafish were severely α-tocopherol-deficient.

### 3.2. Effect of Age on Muscle Metabolites

**Figure 2:**
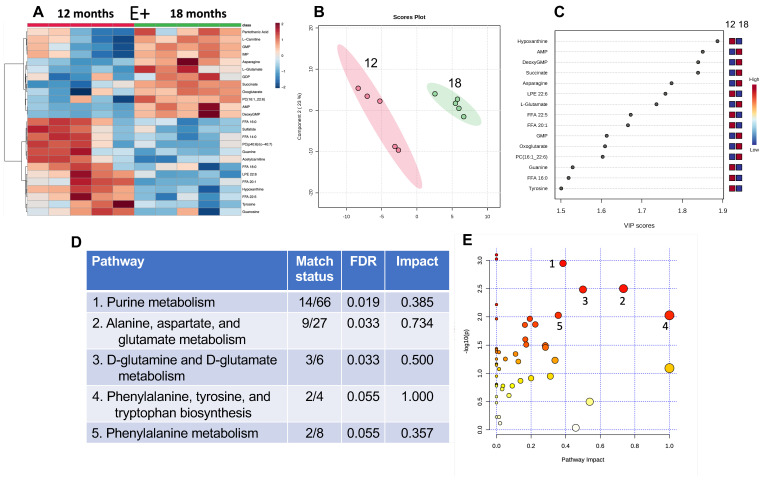
Analysis of E+ fish muscles at 12 vs. 18 months. The heatmap (**A**) shows the top 25 metabolites separating groups and their relative abundances per sample (red represents a higher concentration per sample while blue indicates a lower one). The partial least squares-discriminant analysis (PLS-DA) scores plot (**B**) separates 12- and 18-month E+ muscle samples by feature differences and displays a 95% confidence region around samples. Important features plot displays variable importance in projection (VIP) (**C**) of the PLS-DA and provides an estimation of the discriminatory power of each metabolite with a score. Larger VIP scores indicate greater discriminatory power in the PLS-DA models. The table (**D**) lists the top values shown in the graph of pathway analysis, while figure (**E**) displays pathway analysis by ranking metabolites by *p*-value (shown as −log10(*p*)) and pathway impact referenced against the KEGG (Kiyoto Encyclopedia of Genes and Genomes) Pathway Database. Red indicates highest impacts, clear the lowest, yellow and orange are intermediate.

To evaluate the effect of aging on muscle metabolites, the metabolomics data from muscles of E+ fish at 12 and 18 months are shown in Figure 2. The heatmap shows the comparisons of muscle metabolites from each of the fish in the 12-month compared with the 18-month E+ groups (Figure 2A). PLSDA and VIP scores (Figure 2B,C, respectively) showed that the top four metabolites separating age groups were hypoxanthine, which was increased in the 12-month group, and AMP, 2′-deoxyguanosine 5′-monophosphate (abbreviated in diagram), and succinate, which were higher in the 18-month group.

The VIP scores for lysophosphatidyl-ethanolamine with docosahexaenoic acid (DHA, LPE 22:6) and some free fatty acids (FFA 22:5, 20:1 and 16:0) were lower in the muscles from the 18-month group. Remarkably, a related phospholipid, phosphatidylcholine 16:1_22:6 (PC 16:1_22:6), was higher in the older group.

MetaboAnalyst pathway analysis was used to generate the accompanying table by ranking significance and impact against known KEGG zebrafish biochemical pathways (Figure 2D, and as illustrated in the graph in Figure 2E). Five pathways were identified with low false discovery rates (FDRs). Pathways that were highly impacted with a significant FDR were the purine metabolism pathway; the pathways for alanine, aspartate, glutamate; as well as the pathway for the interconversion of glutamate and glutamine. Metabolites in the pathways for phenylalanine, tyrosine, and tryptophan, as well as for phenylalanine biosynthesis, were also different (FDR < 0.05) between the 12- and 18-month muscle metabolites.

### 3.3. Effect at 12 Months of Vitamin E Deficiency on Muscle Metabolites

**Figure 3:**
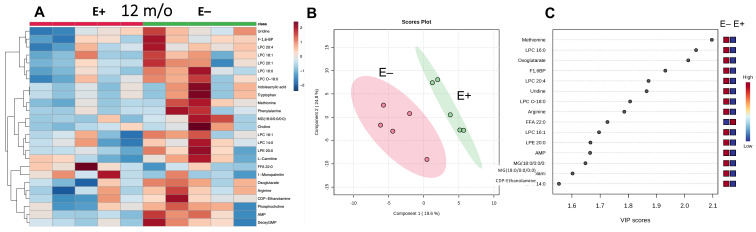
Metabolomic differences between E+ and E− fish muscles at 12 months. Shown are the heatmap (**A**) and PLS-DA scores plot (**B**), which separates E+ and E− muscle samples by feature differences and displays a 95% confidence region around samples and VIPs (**C**). There were insufficient statistically significant differences in the data from muscles of E+ and E− fish at 12 months for statistically meaningful pathway analysis.

The metabolomics data from muscles of E+ compared with E− fish at 12 months are shown in the heatmap (Figure 3A). Further analyses of these data using PLS-DA and VIP scores (Figure 3B,C, respectively) show that all of the top metabolites separating the E+ and E− muscles were elevated in the E− muscles, with the exception of the higher FFA 24:0 in E+ muscles. Higher lysophosphatidylcholines (LPCs) were also observed in E− fish muscles. The elevated lysophospholipids largely contained saturated or monounsaturated fatty acids (16:0, 18:0, 18:1, 20:0), suggesting increased phospholipid turnover and increased metabolism, as has been observed previously in E− embryos [24].

Based on the VIP scores (Figure 3C), some amino acids (methionine and arginine), as well as metabolites related to energy metabolism (oxoglutarate (α-ketoglutarate), AMP, fructose-1,6-biphosphate (F1-6BP)) are higher in the E− group. Notably, fructose-1,6-biphosphate is a key metabolite in the pentose phosphate pathway necessary for the generation of NADP(H). The increases in E− muscle metabolites may reflect increased cellular metabolism to counterbalance the effects of increased lipid peroxidation due to vitamin E deficiency, as also observed in E− embryos [15].

### 3.4. Effect of 18 Months of Vitamin E Deficiency of Muscle Metabolites

**Figure 4:**
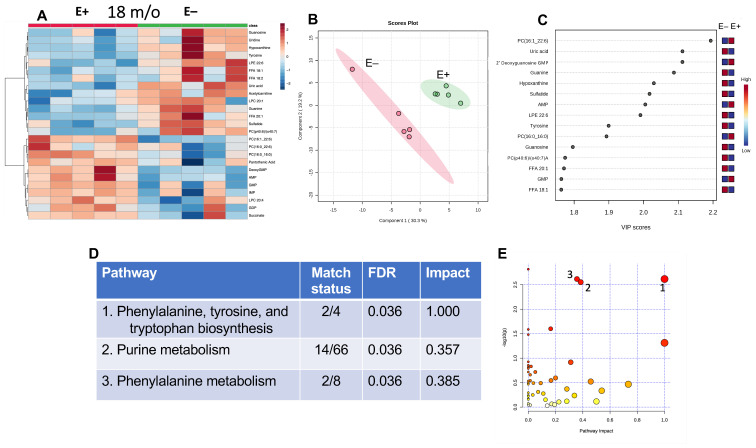
Metabolomic differences between E+ and E− fish muscles at 18 months. Shown are the heatmap (**A**) PLS-DA scores plot (**B**) and VIP scores (**C**). The table (**D**) lists the top values shown in the graph of pathway analysis. (**E**) displays pathway analysis by ranking metabolites by *p*-value (shown as −log10(*p*)) and pathway impact referenced against the KEGG Pathway Database. Red indicates highest impacts, clear the lowest, yellow and orange are intermediate.

To evaluate the effect of prolonged vitamin E deficiency on muscle metabolites in older fish, the E+ and E− groups at 18 months of age were compared (Figure 4). These outcomes showed a large number of purines separating the groups. Of the top 25 metabolites shown in the heatmap (Figure 4A), 8 were purines.

Further analysis of these data using PLSDA and VIP scores (Figure 4B,C, respectively) shows that of the top four metabolites separating the E+ and E− muscles at 18 months, two were higher and two were lower in the E− muscles. PC (16:1_22:6), a major membrane phospholipid, and 2′deoxyguanosine monophosphate were lower in the E− group, while uric acid and guanine were higher (Figure 4C). LPE 22:6 and monounsaturated fatty acids (FFA 20:1 and 18:1) were also elevated in the E− muscles.

**Figure 5:**
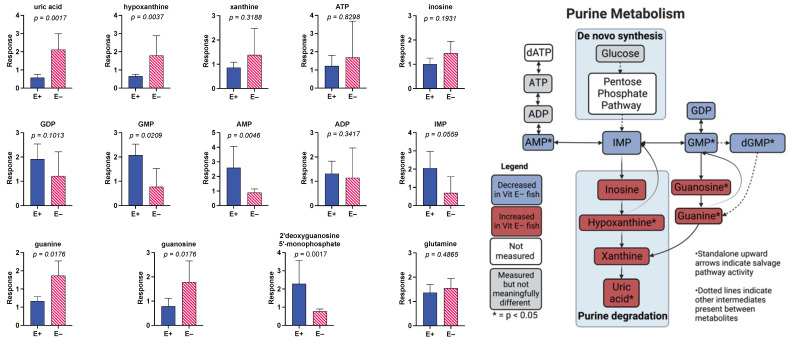
Purine metabolism is altered in E− zebrafish muscle at 18 months. Bar graphs showing Student’s *t*-test of 14 metabolites involved in KEGG pathway of zebrafish purine metabolism. Relative response (mean ± SD, n = 5 per group, E+ (blue bar), E− (red stripped bar), group = 18 months). The right graphic is a visualization of the changes in various purines within the purine metabolism pathway. Right graphic created with BioRender.com.

MetaboAnalyst pathway analysis was used to generate the accompanying table by ranking significance and impact against known KEGG zebrafish biochemical pathways (Figure 4D) and as illustrated in the graph (Figure 4E). Impactful pathway changes included purine metabolism. Significantly different purines (shown in the bar charts, Figure 5) between E+ and E− muscles suggest that purine synthesis in the muscles of E– fish are downregulated while purine degradation is upregulated.

Phenylalanine, tyrosine, and tryptophan biosynthesis with alterations in tyrosine metabolism were also altered significantly.

### 3.5. Effect of Prolonged Vitamin E Deficiency on Muscle Metabolites

**Figure 6:**
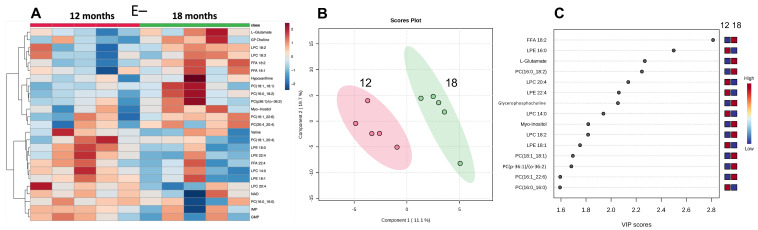
Metabolomic differences between E− fish at 12 and 18 months. Shown are the heatmap (**A**) PLS-DA scores plot (**B**,**C**) VIP scores. There was insufficient statistically significant differences in the data from muscles of E− fish at 12 and 18 months for statistically meaningful pathway analysis.

To evaluate the impact of prolonged vitamin E deficiency, metabolomics data from the muscles of E− fish at 12 and 18 months was examined using a heatmap (Figure 6A). Further analysis using PLSDA and VIP scores were used to evaluate the metabolites separating the E− at 12 and 18 months (Figure 6B,C, respectively). The 18-month E− muscles had higher levels of FFA 18:2 (Linoleic acid), L-Glutamate, and PC (16:0_18:2), while LPE 16:0 was lower. In this comparison, many of the VIP species were lysophospholipids and PCs. Interestingly, PC (16:1_22:6), FFA 18:2, and LPC 18:2 were elevated in the older 18-month muscle samples, suggesting that various compensatory mechanisms were taking place in the 18-month group [17,25].

## 4. Discussion

The purpose of this study was to evaluate the roles of increased oxidative stress and lipid peroxidation caused by vitamin E deficiency on muscle metabolism during aging. We used zebrafish fed defined diets with or without α-tocopherol for 12 or 18 months. Unlike the zebrafish embryo [25], which is a closed system with no input of additional dietary nutrients, the adult zebrafish is fed a macro- and micro-nutrient-sufficient diet, with the exception of vitamin E. Thus, the adult has a continued input of carbohydrates, fats, and proteins, as well as essential nutrients and energy. This adult zebrafish model is much more physiologically relevant to free living organisms.

The 12-month-old E+ fish (Figure 2) had increased levels of AMP and succinate compared with the 18-month-old E+ fish. AMP-activated protein kinase is a marker of dysregulation of energy metabolism [26]. Furthermore, there has been a growing interest in succinate as a marker to signal increased energy needs. Succinate is associated with pathological mitochondrial dysfunction and increased skeletal muscle oxygen consumption [27,28]. We also found evidence of increased lipid peroxidation in the older fish based on the decreases in LPE-DHA and PC-DHA. LPE-DHA is a precursor of PC-DHA via the PEMT pathway [29]. Interestingly, the PC_16:1_22:6 in the muscles from the E+ compared to E− fish were similar at 12 months; however, by 18 months, the E− muscles had higher levels than E− muscles at 12 months, but the muscles in the E+ fish were higher than in the 18-month-old deficient fish. These data suggest that the prolonged vitamin E deficiency and associated lipid peroxidation were depleting PC-DHA by 18 months.

Other pathways that were highly impacted by aging were the purine metabolism pathway; the pathways for alanine, aspartate, and glutamate; the pathway for the interconversion of glutamate and glutamine; as well as the phenylalanine metabolism (Figure 2D,E). Notably, phenylalanine has been reported to be released from muscle during fasting and to regulate mTOR [30], potentially indicative of muscle wasting [31]. Studies evaluating sarcopenia in humans using a metabolomic-based approach have proposed that plasma L-alanine, gluconic acid, proline, and tryptophan could be biomarkers of severe sarcopenia [31].

Interestingly, the dysregulation of phenylalanine metabolism has also been observed in aging humans and has been reported in several studies [32,33,34]. Aging may also increase the requirement for glutamate, since this amino acid is needed for glutathione synthesis (a tripeptide consisting of glutamate, cysteine, and glycine). Notably, glutathione is a critical water-soluble antioxidant and is also involved in the regeneration of the antioxidant capacity of vitamin E [35,36]. Unfortunately, glutathione, and furthermore, malondialdehyde (MDA), was not specifically measured in this experiment due to strict requirements for sample isolation and storage. Previous studies in vitamin E-deficient adult zebrafish have documented increased MDA in the brain [12] and in the liver [22], supporting the likelihood of increased lipid peroxidation in these fish.

Examination of 18-month-old fish with or without vitamin E deficiency emphasized the possibility of hormesis (Figure 4 and Figure 5); that is, the prolonged vitamin E deficiency potentiated metabolic salvage pathways. Specifically, alterations in various purines between E+ and E− muscles suggest that purine synthesis in the muscles of E− fish are downregulated, while purine degradation is upregulated. Additionally, the higher uric acid in E− muscles provides a means to potentiate antioxidant defenses because uric acid can function as a water-soluble antioxidant [37]. Unlike humans and other primates [38], zebrafish [39] express the gene that encodes uricase, an enzyme that converts uric acid to allantoin [40]. Previous studies have shown that allantoin is elevated in vitamin E-deficient, non-primate animals [41,42]. Interestingly, in humans, an alternative pathway of allantoin production is the free-radical-mediated oxidation of uric acid [43]. Although uric acid was elevated, allantoin was not detected in the E− fish. Similar increases in other purines, such as inosine and hypoxanthine, have also been reported during exercise-mediated stress [44].

Previously, we reported increased IMP and AMP levels, as well as oxidative stress, in zebrafish fed a diet deficient in both vitamin E and ascorbic acid for a prolonged period [45]. While ascorbic acid was provided in the diet in the present study, it may have undergone increased utilization. Ascorbic acid can be oxidized during redox reactions between peroxyl radicals generated during lipid peroxidation and α-tocopherol scavenging [46]. This “antioxidant network” consists of various antioxidants, including ascorbic acid, glutathione, and NADPH, and may be subject to other factors and pathways, including oxidative stress response, purines, and energy metabolism, such as AMPK activity [45,47].

As expected, based on its peroxyl radical scavenging capability, when assessing vitamin E sufficiency and deficiency, the metabolomic differences between E+ and E− muscles at 12 months showed likelihood of increased membrane lipid peroxidation based on increases in lysophospholipids, many with saturated fatty acids (Figure 3). Similarly, the E− muscles at 12 compared with 18 months again showed similar impacts of lipid peroxidation on phospholipid metabolism. An increased longevity of the feeding period for all groups, but particularly the E+ vs. E− comparisons, may yield more striking results.

## 5. Conclusions

The use of metabolomics allows for the measurement of a great number of metabolites but only provides limited semi-quantitative analyses [48]. The purpose of this type of evaluation is to generate future hypotheses. This study provides some provocative observations for future experiments. We found that aging and vitamin E deficiency for 18 months caused markedly different disruptions to metabolic function. Comparison of metabolite changes in muscle following consumption of a vitamin E-sufficient compared with a vitamin E-deficient diet for 12 months in zebrafish showed relatively few statistically striking results. However, some evidence of lipid peroxidation and increased phospholipid turnover was present in the E– muscles at 12 months, along with increased concentrations of metabolites related to energy metabolism such as AMP, α-ketoglutarate, and fructose 1,6 biphosphate. These data are similar to those in embryos from vitamin E-deficient zebrafish [15,16,49].

The metabolomics data from the muscles of E− fish at 12 compared with 18 months showed that the 18-month E− muscles had higher levels of FFA 18:2 (Linoleic acid), L-Glutamate, and PC (16:0_18:2), while LPE 16:0 was lower. In this comparison, many of the VIP species were lysophospholipids and PCs. Interestingly, PC (16:1_22:6) at 18 months in the E− muscles was lower than that in the 18-month E+ muscles, as discussed above.

Both aging and vitamin E deficiency at 18 months caused significant metabolic pathway disruptions (Figure 4 and Figure 5). The largest change caused by either aging or vitamin E deficiency was a disruption in purine metabolism. We found that aging tended to demonstrate decreased purine degradation while vitamin E deficiency showed the opposite, an upregulated purine degradation. The increase in purine degradation metabolites during vitamin E deficiency, particularly uric acid, is of interest because uric acid may act as an antioxidant. Aging was associated with energy dysregulation and increased lipid peroxidation, especially those lipids containing DHA. Changes were also observed during aging in several amino acids that have previously been reported by others during aging, muscle wasting, or sarcopenia. Vitamin E deficiency seemed to have major effects on phospholipid metabolism, but aging had additional impacts on purine metabolism. In sum, both aging and vitamin E deficiency at 18 months demonstrate significant alterations within essential pathways, yet sufficient differences, such as the directionality of regulation, remain that appear to differentiate aging and the dietary intervention. Remarkably, vitamin E deficiency seemed to increase the resilience of the 18-month-old animals to upregulate protective mechanisms. Whether other factors such as hormesis are at play and if there are further associated physiologic implications, such as muscle degeneration or loss of strength, will require future studies. An alternative explanation could be that the animals that could not survive both aging and vitamin E deficiency died, and what we observed is a survivorship bias. Further studies are needed to explore these possibilities.

## Data Availability

Data is contained within the manuscript and supplementary materials.

## Supplementary Materials

The following supporting information can be downloaded at: https://www.mdpi.com/article/10.3390/antiox12061160/s1, Table S1: Zebrafish muscle metabolites.
